# Primary extraovarian adult granulosa cell tumor of the greater omentum: a case report and literature review

**DOI:** 10.3389/fonc.2025.1689815

**Published:** 2026-01-08

**Authors:** Ying Zeng, Liang Lv, Liping Luo

**Affiliations:** 1 Department of Pathology, The Thirteenth People’s Hospital of Chongqing, Chongqing, China; 2 Department of Pathology, Chongqing Public Health Medical Center, Chongqing, China; 3 Department of Pathology, Daping Hospital, Army Medical University, Chongqing, China

**Keywords:** extraovarian, adult granulosa cell tumor, omentum, sex cord-stromal tumor, treatment

## Abstract

Extraovarian adult granulosa cell tumors are rare. The diagnosis of extraovarian adult granulosa cell tumors is challenging. Here we presented a case of extraovarian adult granulosa cell tumor originating in the greater omentum. Along with a review of the literature, we aim to summarizes its clinicopathological features to enhance recognition of this tumor. A 55-year-old female patient presented with dull abdominal pain for 3 days. Abdominal CT revealed a soft tissue density mass in the right lower abdomen, measuring 9.0 × 6.3 × 6.0 cm. The mass demonstrated an regular contour and indistinct borders, with heterogeneous density. Histopathological examination revealed a tumor encapsulated by a fibrous capsule. The tumor cells were arranged in cords and trabeculae with a streaming pattern. Call-Exner bodies were identified. The tumor was composed of round, oval, or polygonal cells. Nuclear grooves and “coffee-bean” nuclei were observed. The mitoses figures was 1 mitoses per 10 high-power fields (HPFs, 40×objective, field area = 0.237 mm²). Tumor cells were positive for α-inhibin, SF-1, FOXL2, WT-1, and Vimentin. The Ki67 proliferation index was 20%. The histological morphology and immunophenotype support the diagnosis of adult granulosa cell tumor. Furthermore, intraoperative abdominal exploration and imaging studies revealed no lesion in the uterus or bilateral adnexa. Therefore, the diagnosis of an extraovarian adult granulosa cell tumor was established. Extraovarian granulosa cell tumors occur predominantly in middle-aged women, most commonly in the retroperitoneum, and typically present as large cystic-solid masses. The diagnosis of extraovarian granulosa cell tumor requires the definitive exclusion of a primary ovarian tumor. Diagnosis primarily relies on characteristic histomorphological features supported by immunohistochemical staining, notably positivity for markers such as α-inhibin. In diagnostically challenging cases, *FOXL2* mutation testing can serve as a valuable ancillary tool to confirm the diagnosis. Surgical resection is the mainstay of treatment in most cases. Consequently, we recommend that all patients with primary extra-ovarian granulosa cell tumors enter a protocol of long-term surveillance, including periodic imaging and hormonal marker assessment.

## 1 Introduction

Ovarian granulosa cell tumors (GCTs) are uncommon, low-grade malignancies thought to originate from the gonadal ridge mesenchyme, accounting for approximately 2%-5% of all ovarian neoplasms. They are classified into juvenile and adult types (1). While they occur predominantly in the ovary, rare cases have been documented at extraovarian sites, including the broad ligament, retroperitoneum, and mesentery, with a total of 29 cases reported in the literature (2–30). To date, only a single case of a primary extraovarian adult granulosa cell tumor (AGCT) arising in the greater omentum has been described (22).

The pathogenesis of extraovarian granulosa cell tumors remains incompletely elucidated. The predominant hypothesis suggests an origin from ectopic gonadal tissue displaced along the embryonic migratory pathway of the gonadal ridge. An alternative theory proposes derivation from the coelomic epithelium or mesonephric (Wolffian) remnants, which could account for their occurrence at the various documented sites (21). This etiological uncertainty, combined with nonspecific clinical presentation and histological resemblance to more common entities like epithelial ovarian carcinomas or sarcomas, frequently leads to diagnostic challenges and delays.

Moreover, due to the extreme rarity of omental AGCTs, diagnostic workup, and optimal management strategy are poorly characterized. This report aims to address this knowledge gap by presenting a unique case of a primary extraovarian AGCT in the greater omentum of a postmenopausal woman. This case provides several critical insights: the tumor can develop without concurrent uterine or adnexal lesions; its clinical symptoms and hormone levels can be entirely nonspecific; and its diagnosis relies on histopathological and immunohistochemical features consistent with its ovarian counterpart. Furthermore, we summarizes its clinicopathological features to enhance recognition of this tumor with a literature review.

## 2 Methods

### 2.1 Clinical timeline and management

**Figure 1:**
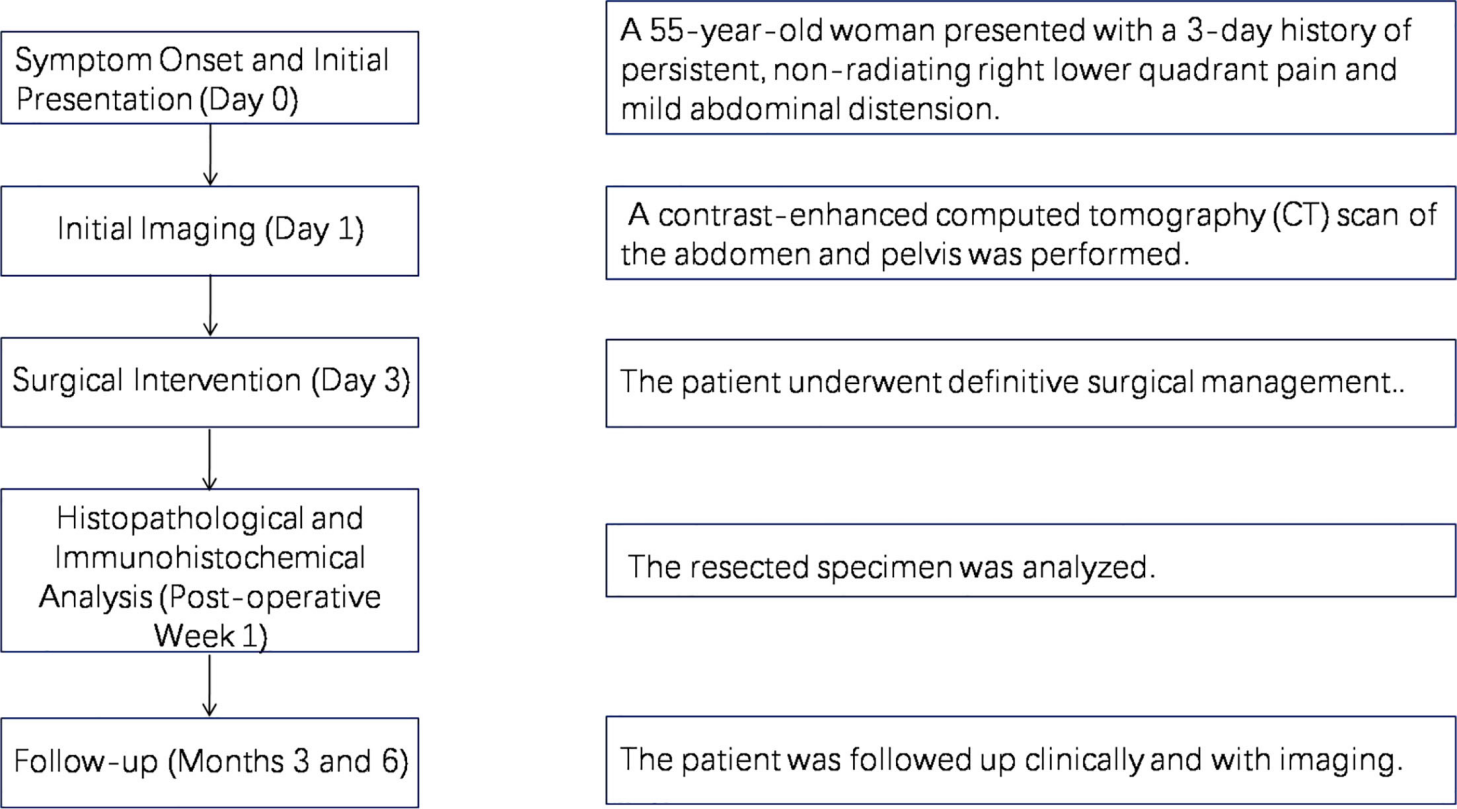
The patient’s diagnostic and therapeutic pathway.

The patient’s diagnostic and therapeutic pathway is summarized in **Figure 1** and followed this chronological sequence:

#### 2.1.1 Symptom onset and initial presentation (day 0)

A 55-year-old woman presented with a 3-day history of persistent, non-radiating right lower quadrant pain and mild abdominal distension.

#### 2.1.2 Initial imaging (day 1)

A contrast-enhanced computed tomography (CT) scan of the abdomen and pelvis was performed.

#### 2.1.3 Surgical intervention (day 3)

The patient underwent definitive surgical management.

#### 2.1.4 Histopathological and immunohistochemical analysis (post-operative week 1)

The resected specimen was analyzed.

#### 2.1.5 Follow-up (months 3 and 6)

The patient was followed up clinically and with imaging.

### 2.2 Radiological investigation

The CT examination was conducted on a 256-slice scanner (Siemens Somatom, Definition Flash). The protocol included non-contrast, arterial (30-second delay), and portal venous (70-second delay) phases following intravenous administration of iodinated contrast. Acquisition parameters were: 120 kV, automated tube current modulation (SD 25), slice thickness 0.625 mm. All images were independently interpreted by a fellowship-trained abdominal radiologist with 15 years of experience. The absence of involvement of the adjacent small bowel, liver, and major vessels was confirmed based on the lack of stranding, mass effect, or abnormal enhancement in these structures on multiplanar reconstructions.

### 2.3 Surgical procedure and assessment

Laparoscopic Exploration and Laparoscopic Tumor Resection were performed. Criteria for radicality (R0 Resection): Macroscopic complete resection with intent to achieve a microscopically negative margin (R0) was the objective. This required a grossly negative margin of >1 cm from the tumor and en-bloc removal of all pertinent lymphatic tissue. Intraoperative Assessment: Systematic inspection of the peritoneal cavity, liver surface, pelvis and uterus with fallopian tubes and ovaries, regional lymph node basins was conducted. Peritoneal lavage cytology was performed and the resection specimen was submitted for histopathological evaluation. Resection Status: The final margin status was confirmed postoperatively by pathology.

### 2.4 Histopathological and immunohistochemical analysis

The specimen was fixed in 10% neutral buffered formalin for 24 hours. A total of 16 representative tissue blocks were embedded, from which 4μm sections were cut and stained with hematoxylin and eosin. Mitotic activity was assessed in 50 consecutive high-power fields (HPF) at 40×magnification (field area 0.237 mm²) and expressed as mitoses per 10 HPF. The Ki-67 proliferation index was determined by manual count of at least 1000 tumor cells in the area of highest nuclear labeling (hotspot method).

Immunohistochemistry was performed on a Ventana Benchmark XT autostainer using the following antibody panel detailed in **Table 1**. Appropriate positive and negative controls were run concurrently.

**Table 1:** Immunohistochemical profile of the tumor.

Antibody	Result	Staining pattern	Interpretation	Antibody clone number and manufacturer
a-inhibin	Positive	Strong,diffuse nucleus/membranous/cytoplasmic	Supports diagnosis	AMY82 (Mouse Monoclonal), Zhongshan Golden Bridge
SF-1	Positive	Strong,diffuse nucleus	Supports diagnosis	EP434 (Rabbit Monoclonal), Maixin
FOXL2	Positive	Strong,diffuse nucleus	Supports diagnosis	B65(Rabbit Monoclonal), Zhongshan Golden Bridge
WT-1	Positive	Strong,diffuse nucleus	Supports diagnosis	OTIRIH(Rabbit Monoclonal), Zhongshan Golden Bridge
Vimentin	Positive	Strong,diffuse cytoplasmic	Supports diagnosis	MX034(Rabbit Monoclonal), Maixin
CD10	Negative	N/A	Supports diagnosis	UMAB235(Mouse Monoclonal), Zhongshan Golden Bridge
D2-40	Negative	N/A	Supports diagnosis	D2-40 (Mouse Monoclonal),Maixin
SMA	Focal positivity	Strong,cytoplasmic	Supports diagnosis	IA4 (Mouse Monoclonal),Maixin
Calretinin	focal weak positivity	N/A	Supports diagnosis	MX027(Mouse Monoclonal),Maixin
CD117	Negative	N/A	Used to exclude certain diseases (e.g., gastrointestinal stromal tumor	Rabbit IgG, YR145,Maixin
CD34	Negative	N/A	Used to exclude certain diseases (e.g., vascular tumor)	QBEnd/10(Mouse Monoclonal),Maixin
Desmin	Negative	N/A	Used to exclude certain diseases (e.g., myogenic tumor)	MCO4b(Mouse Monoclonal),Maixin
Dog-1	Negative	N/A	Used to exclude certain diseases (e.g., gastrointestinal stromal tumor)	SP31(Mouse Monoclonal),Maixin
S-100	Negative	N/A	Used to exclude certain diseases (e.g., malignant melanoma, neurogenic Tumor)	Rabbit pAb, Zhongshan Golden Bridge
SDHB	Negative	N/A	Used to exclude certain diseases (e.g.,SDHB deficient tumor)	OTI1H6 (Mouse Monoclonal),Zhongshan Golden Bridge
STAT6	Negative	N/A	Used to exclude certain diseases (e.g.,solitary fibrous tumor)	EP325(Rabbit Monoclonal), Maixin
Arginase1	Negative	N/A	Used to exclude certain diseases (e.g.,Hepatocellular carcinoma)	EP261 (Rabbit Monoclonal), Zhongshan Golden Bridge
Glypican3	Negative	N/A	Used to exclude certain diseases (e.g.,Hepatocellular carcinoma)	MAXIM001(Mouse Monoclonal),Maixin
BAP1	retained	N/A	Used to exclude certain diseases (e.g.,Mesothelioma)	C-4(Mouse Monoclonal), Jiehao
keratin20	Negative	N/A	Used to exclude certain diseases (e.g.,metastatic carcinoma)	EP23 (Rabbit Monoclonal), Zhongshan Golden Bridge
keratin5/6	Negative	N/A	Used to exclude certain diseases (e.g.,Metastatic carcinoma)	RM341(Rabbit Monoclonal), Zhongshan Golden Bridge
keratin7	Negative	N/A	Used to exclude certain diseases (e.g.,metastatic carcinoma)	UMAB262(Mouse Monoclonal),Zhongshan Golden Bridge
HMB45	Negative	N/A	Used to exclude certain diseases (e.g.,metastatic melanoma)	HMB 45(Mouse Monoclonal),Maixin
PAX8	Negative	N/A	Used to exclude certain diseases (e.g.,Metastatic ovarian carcinoma, renal cell carcinoma)	EP298 (Rabbit Monoclonal), Maixin
Synaptophysin	Negative	N/A	Used to exclude certain diseases (e.g.,Metastatic neuroendocrine carcinoma)	MOX38(Mouse Monoclonal),Maixin
Thyroglobulin (TG)	Negative	N/A	Used to exclude certain diseases (e.g.,metastatic carcinoma of thyroid)	0TI8F2(Mouse Monoclonal),Zhongshan Golden Bridge
TTF1	Negative	N/A	Used to exclude certain diseases (e.g.,carcinoma of thyroid and lung)	SP724(Mouse Monoclonal),Maixin
Villin	Negative	N/A	Used to exclude certain diseases (e.g.,carcinoma of digestive tract)	CWWB1(Mouse Monoclonal),Maixin
Ki67	0.2	Strong, nucleus	Detection of tumor proliferation activity	UMAB107 (Mouse Monoclonal),Zhongshan Golden Bridge

### 2.5 Follow-up

The patient was followed at 3-month intervals for the first year with clinical examination and CT imaging. Follow-up data is current through July 2025.

### 2.6 Narrative review methodology

A narrative review of the literature was conducted in accordance with the Scale for the Assessment of Narrative Review Articles guidelines. A narrative literature review was conducted with a primary focus on publications from the last decade to reflect contemporary diagnostic and clinical paradigms. However, given the extreme rarity of primary extraovarian granulosa cell tumors, all historically reported cases were included in the clinicopathological summary to ensure a complete analysis of the known spectrum of this disease. Literature retrieval was performed using the PubMed and Web of Science databases, covering publications from the inception of each database to June 30, 2025 using the keywords: “[extraovarian]” “[granulosa cell tumor],” Inclusion criteria were: (1) surgically and pathologically confirmed, (2) case reports or series and (3)English full-text articles. Exclusion criteria were: (1) non-English articles, (2)Ovarian involvement, (3)the lack of pathological confirmation, and (4)the unavailability of full-text articles. Key data from the 29 included studies are summarized in **Table 2**, which contains the following fields: reference, number of patients, age, diagnosis, intervention, outcome, and duration of follow-up.

**Table 2:** Summary of clinicopathological characteristics of primary extraovarian granulosa cell tumor.

Reference	case	Age (years)	location	Size(cm)	Clinical signs and symptoms	Imaging examination	Hormone level and tumor marker tests	Immunohistochemistry	Molecular detection	Pathological diagnosis	Whether ovarian lesions have been ruled out	Whether to remove it surgically	Chemotherapy or radiotherapy	Follow-up time/result
Voigt WW(2)1938	1	51	Retroperitoneum	NA	Abdominal distension	An X-ray examination was not performed due to cost issues.	NA	NA	NA	Adult exgranular cell tumor	Yes	Yes	NA	NA
Ragins AB(3)1940	2	37	Retroperitoneum	NA	NA	NA	NA	NA	NA	Adult exgranular cell tumor	Yes	Yes	NA	NA
Powell C(4)1940	3	30	Retroperitoneum	NA	NA	NA	NA	NA	NA	Adult exgranular cell tumor	Yes	Yes	NA	NA
Reddy DB(5)1963	4	40	Retroperitoneum	NA	NA	NA	NA	NA	NA	Adult exgranular cell tumor	Yes	Yes	NA	NA
Orselli RC(6)1973	5	52	adrenal gland	NA	Abdominal distension	NA	NA	NA	NA	Adult exgranular cell tumor	Yes	Yes	NA	NA
Keitoku M(7)1997	6	45	Broad ligament、Retroperitoneum	NA	Abdominal distension and pain	Solid tumor with multiple nodules	The preoperative estradiol level was 1029pg/ml, and the postoperative estradiol level was less than 20pg/ml. The tumor markers (CA199, CA125, CEA, AFP, squamous cell carcinoma antigen) were negative.	α-inhibin(+), EMA(-)	Not detected	Adult exgranular cell tumor	Yes	Yes	NA	36months/ recurrence
Robinson JB(8)1999	7	67	The lateral wall of the right pelvic cavity	15	Diffuse tenderness in the lower right abdomen	The tumor is cystic and solid with an unclear boundary	NA	α-inhibin, ER, PR, Vimentin(+),S100(Focal weak+),Syn, CgA, CK(-)	Not detected	Adult exgranular cell tumor, endometrial curettage shows simple hyperplasia accompanied by focal glandular complex hyperplasia	Yes	Yes	NA	NA
Hameed A(9)2000	8	69	The right adrenal gland	9	Irregular uterine bleeding	Solid tumor with hemorrhage	NA	α-inhibin(+)	Not detected	Adult exgranular cell tumor, endometrial curettage shows simple hyperplasia.	Yes	Yes	NA	NA
Kim SH(10)2001	9	54	Retroperitoneum	8.8	Bleeding after sexual intercourse	Lobulated solid tumor with necrotic areas	Hormone tests were not conducted. Tumor markers (CA199, CEA) were negative, but CA125 increased to 83.08U/ml	α-inhibin(-), Vimentin(Focal weak+),EMA, NSE, Syn(-)	Not detected	Adult exgranular cell tumor	Yes	Yes	NA	NA
Paul PC(11)2009	10	58	Retroperitoneum、Mesentery	16	Pain in the right abdomen	The tumor had a clear boundary, cystic and solid, accompanied by necrosis and hemorrhage	NA	α-inhibin(+),EMA(-)	Not detected	Adult exgranular cell tumor	Yes	Yes	NA	NA
Manjiri R N(12) 2010	11	54	Mesentery	13	Acute abdominal pain	The tumor boundary is irregular, cystic and solid with hemorrhage	No hormone test was conducted	α-inhibin(+),EMA(-)	Not detected	Adult exgranular cell tumor	Yes	Yes	NA	NA
Al-Shraideh Y(13)2012	12	64	Retroperitoneum	13	NA	The tumor is lobulated with cystic regions.	NA	α-inhibin(+),EMA(-)	Not detected	Adult exgranular cell tumor	Yes	Yes	NA	NA
Soydinc HE(14)2012	13	22	Right pelvic cavity, behind the right pubic bone	8	Pelvic pain, menstrual disorders	Cystic and solid tumor	Inhibin levels are normal, and tumor markers (CA199, CA125, CA153, AFP, CEA) are negative.	NA	Not detected	Adult exgranular cell tumor	Yes	Yes	NA	NA
Barbosa LCR(15)2013	14	62	Left fallopian tube	6	NA	Complex cystic tumors	NA	α-inhibin, CR(+),EMA(-)	Not detected	Adult exgranular cell tumor	Yes	Yes	NA	NA
Chiriac D(16)2014	15	50	Retroperitoneum	6.5	Diffuse pelvic pain	Multilocular cystic and heterogeneous tumor	NA	NA	Not detected	Adult exgranular cell tumor	Yes	Yes	Cell inhibition therapy was received after the operation.	24 months/ No recurrence
Rifki JS(17)2016	16	52	Retroperitoneum	8	NA	Cystic and solid tumor	NA	α-inhibin, CR, Vimentin(+),EMA(-)	Not detected	Adult exgranular cell tumor	Yes	Yes	NA	NA
Medhi P(18)2016	17	60	Retroperitoneum	11	NA	The tumor boundary is clear.	NA	α-inhibin(+),EMA(-)	Not detected	Adult exgranular cell tumor	Yes	Yes	NA	NA
Vasu PP(19)2016	18	69	Retroperitoneum	10	Abdominal discomfort	Cystic and solid tumor	NA	α-inhibin(+),EMA(-)	Not detected	Adult exgranular cell tumor	Yes	Yes	NA	NA
Singh A(20)2019	19	58	Broad ligament	11	NA	Pelvic tumor	NA	α-inhibin, CR(+),CK(-)	Not detected	Adult exgranular cell tumor	Yes	Yes	NA	NA
Vassallo MC(21)2019	20	63	adrenal gland	9	NA	Tumor of the right adrenal gland	NA	NA	Not detected	Adult exgranular cell tumor	Yes	Yes	NA	NA
Swain SK(22)2020	21	55	Greater omentum	7	NA	Cystic and solid tumor	NA	α-inhibin(+),CK(-)	Not detected	Adult exgranular cell tumor	Yes	Yes	NA	NA
Machado I(23)2020	22	57	Retroperitoneum	5	Pain and fever in the right costal region and right groin region (38.2)	Cystic solid tumor with hemorrhage, with a clear boundary	NA	α-inhibin, CR, S100, PR, WT1, CD99(+), CK7, CK20, CgA, Syn, CDX2, EMA, TTF1, MITF, HMB45(-),Ki67(14%+)	*FOXL2* mutation	Adult exgranular cell tumor	Yes	Yes	NA	NA
User İR(24)2021	23	3.5	Retroperitoneum	12	constipation,asymmetrical left upper abdominal distention and palpable immobile mass.	Multiseptate, multiloculated heterogeneous mass with calcifications	Hormone tests showed negative results for serum AFP, beta-hCG, ferritin and urine VMA, HVA. 1.postoperative day 7:Inhibin B (16 pg/ml), FSH (1.76 mU/ml), LH (0.01 mIU/ml) and estrogen (18 pg/ml) levels were in normal limits. 2.The first month:US was normal.	α-inhibin, WT1(80% of tumor cells+),PGP9.5, CD56(50% of tumor cells+),CR, Desmin, HMWCK, AE1/AE3(cells focally+),SALL4, Glypican3, Melan A(-),Ki67(70%+)	Not detected	Juvenile granulosa cell tumor	Yes	Yes	NA	NA
Sharma P(25)2022	24	55	Retroperitoneum	20	There has been intermittent pain in the left lumbar vertebrae for a year	Cystic and solid tumors with hemorrhage and necrosis	Hormone tests showed negative results for β-HCG and tumor markers (AFP, CA125, CEA)	α-inhibin, CR, PR, WT1(+),EMA(-)	Not detected	Adult exgranular cell tumor	Yes	Yes	NA	NA
Yanagawa N(26)2022	25	86	Mesentery	10	Abdominal pain	Solid tumor with hemorrhage and necrosis	The laboratory analysis results showed no abnormalities (no specific test items were mentioned).	α-inhibin, SF1, CR, Melan-A, NCAM, WT1, ER(+), CgA, Syn, EMA, CK7(-)	*FOXL2* mutation	Adult exgranular cell tumor	Yes	Yes	NA	NA
Abedin Y(27)2022	26	64	Retroperitoneum	NA	Intermittent abdominal pain	Multiple abdominal tumors	The preoperative inhibin level was >1300pg/ml, and the postoperative inhibin level was 388pg/ml.	α-inhibin, PR, ER(+)	Not detected	Adult exgranular cell tumor	Yes	Yes	Chemotherapy only: 7 sessions of carboplatin + paclitaxel.	NA
Menon A(28)2023	27	66	Retroperitoneum	21	Abdominal distension, painless abdominal tumor	Cystic tumor	No hormone tests were conducted, and tumor markers (CA125, CEA) were negative.	α-inhibin(+), EMA(-)	Not detected	Adult exgranular cell tumor	Yes	Yes	NA	NA
Oqbani K(29)2023	28	66	Right fallopian tube	1.5	Intermittent abdominal pain of unknown cause	The tumor has unclear boundaries and an irregular shape.	No hormone tests were conducted, and the tumor markers (CA199, CA125, CEA) were negative.	α-inhibin, CR(+), EMA, Syn(-)	Not detected	Adult exgranular cell tumor	Yes	Yes	NA	NA
Moctar NF(30)2024	29	40	Retroperitoneum	21.5	Abdominal distension and diffuse tenderness	The tumor boundary is clear and lobulated.	No hormone tests were conducted, and the tumor markers (CA199, CA125, CEA) were negative.	NA	Not detected	Adult exgranular cell tumor	Yes	Yes	NA	NA
The present case	30	55	Greater Omentum	9	Dull pain in the abdomen	The tumor has clear boundary and an irregular shape	Preoperative and postoperative hormone level tests (progesterone, testosterone, estradiol, prolactin, follicle-stimulating hormone, luteinizing hormone) all showed normal.	α-inhibin, SF-1, FOXL2, WT-1, Vimentin(+),CD10, D2-40, SMA(A few tumor cells+),CR(Scattered weak+),Ki-67(15%+)	Not detected	Adult exgranular cell tumor	Yes	Yes	NA	6 months/ No recurrence

NA, Not Available.

## 3 Results

### 3.1 Clinical and imaging findings

#### 3.1.1 Clinical presentation

A 55-year-old female patient presented with persistent dull abdominal pain for 3 days, without precipitating factors. The pain was accompanied by mild abdominal distension but did not radiate to the shoulder. The patient denied nausea, vomiting, chills, fever, cough, sputum production, chest tightness, shortness of breath, dyspnea, dizziness, headache, urinary frequency, urinary urgency, and dysuria.

#### 3.1.2 Medical history

The patient’s medical history was significant for Hepatitis B and Rheumatoid Arthritis, both diagnosed three decades ago. She had also undergone a laparoscopic appendectomy at another hospital thirty years prior. Her menstrual cycle was regular.

#### 3.1.3 Family history

Her family history was non-contributory for similar or inherited conditions.

#### 3.1.4 Psychosocial history

The patient had no history of smoking or alcohol use. No history of substance abuse was noted.

Prior to this presentation, the patient had not undergone any diagnostic procedures (such as endoscopy or imaging) for these symptoms.

#### 3.1.5 Physical examination

Vital signs were within normal limits: blood pressure 127/76 mmHg, heart rate 91 beats per minute, respiratory rate 20 breaths per minute, and temperature 36.5°C. The cardiorespiratory examination was unremarkable. The abdomen was flat with normal diaphragmatic excursion. No abdominal wall venous distention, visible peristalsis, or hernias were noted. Tenderness was present in the lower abdomen, most pronounced in the right lower quadrant with mild rebound tenderness and localized guarding. No palpable masses, organomegaly, or shifting dullness was detected. Bowel sounds were normoactive. Digital rectal examination (left lateral position): The perianal anatomy was normal. No rectal masses were identified, and the examination glove was free of blood upon withdrawal.

### 3.2 Laboratory findings

Laboratory investigations revealed results as follows. Complete Blood Count: All values were within normal institutional ranges, specifically: Hemoglobin 111 g/L (reference range:115–150 g/L), Platelets 183 ×10^9/L (reference range: 125-350 ×10^9/L, PT 11.0 s (reference range: 9.4-13.8 s), CRP 12.10 mg/L (reference range: 0–8 mg/L). Pre-operative laboratory evaluation demonstrated a normal hormonal profile, specifically: Luteinizing Hormone(LH) 27.79 mIU/ml (reference range: Follicular Phase: 2.12-10.89 mIU/ml, Ovulatory Phase: 19.18-103.03 mIU/ml, Luteal Phase: 1.20-12.86), Follicle-Stimulating Hormone(FSH) 39.06 mIU/ml (reference range: Follicular Phase: 3.85-8.78 mIU/ml, Ovulatory Phase: 4.54-22.51 mIU/ml, Luteal Phase: 1.79-5.12 mIU/ml), Progesterone 0.23 ng/ml (reference range: Mid-Follicular Phase: 0.31-1.52 ng/ml, Mid-Luteal Phase: 5.16-18.56 ng/ml), Estradiol (E2) <20.00 pg/ml (reference range: Early Follicular Phase: 15.16 - 127.81 pg/ml, Mid Follicular Phase: 19.86-148.13pg/ml, Ovulatory Peak: 29.42 -442.62pg/ml, Mid Luteal Phase: 30.34-274.24pg/ml, Postmenopausal: <38.90 pg/ml), Prolactin(PRL) 9.02 ng/ml (reference range: 2.74-19.64 ng/ml), Testosterone <0.10 ng/ml (reference range: 0-0.75 ng/ml). Pre-operative laboratory evaluation demonstrated normal levels of tumor markers, specifically: CA125 19.10 U/ml (reference range: <35.00 U/ml), Carcinoembryonic Antigen (CEA): 2.1 ng/mL (reference range: <5.0 ng/mL), Carbohydrate Antigen 19-9 (CA19-9): 15 U/mL (reference range: <37 U/mL).

### 3.3 Imaging findings

**Figure 2:**
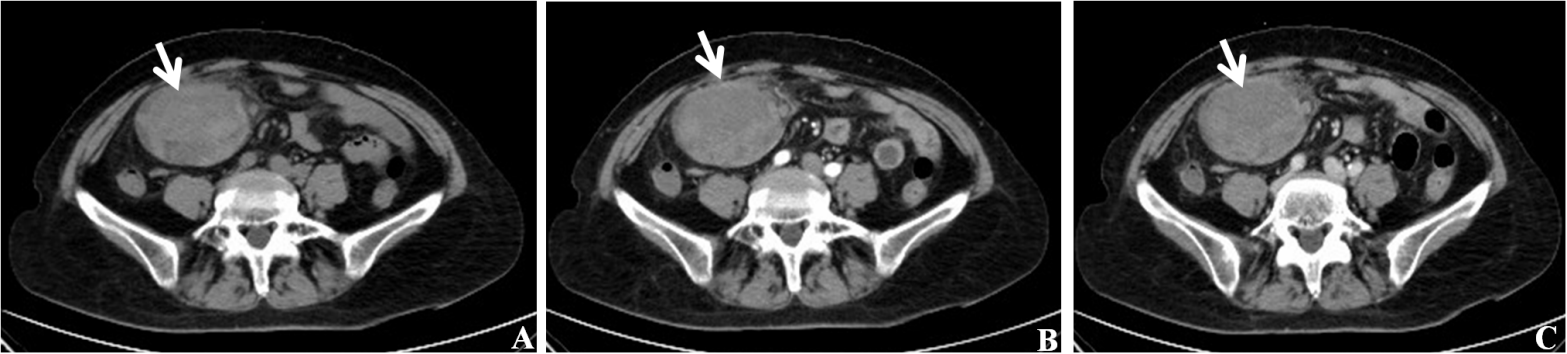
Radiographic characteristics of the extraovarian adult granulosa cell tumor of the greater omentum. **(A)** Abdominal CT scan demonstrated a soft tissue mass within the right abdominal cavity(indicated by the white arrow). The mass exhibited regular and indistinct border and heterogeneous density, containing irregular patchy areas with mild hyperdensity. **(B, C)** Post-contrast images demonstrate heterogeneous enhancement of the lesion(indicated by the white arrow), while the previously noted hyperdense areas exhibit no significant enhancement. These findings were suggestive of a neoplastic lesion with associated hemorrhage. **(B)** Arterial Phase, **(C)** Venous Phase.

Abdominal CT revealed a soft tissue density mass in the right lower abdomen, measuring 9.0×6.3×6.0cm. The mass demonstrated an regular contour and indistinct borders, with heterogeneous density, containing irregular patchy areas with mild hyperdensity (**Figure 2A**). Post-contrast images(Arterial Phase and Venous Phase) demonstrate heterogeneous enhancement of the lesion, while the previously noted hyperdense areas exhibit no significant enhancement (**Figures 2B, C**). These findings were suggestive of a neoplastic lesion with associated hemorrhage. Gastrointestinal stromal tumor (GIST) or Peritoneal tumor was suspected.

### 3.4 Surgical and pathological correlation

During the subsequent laparoscopic procedure, the tumor was identified within the greater omentum of the right lower abdomen, measuring approximately 9.0 × 8.0 × 7.0 cm. This minor discrepancy from the radiographic size was attributed to tumor deformation. It had a firm consistency and was adherent to the abdominal wall. No nodules were observed on the abdominal wall. Examination revealed no abnormalities of the liver, gallbladder, stomach, duodenal bulb, small intestine, colon, upper rectum, uterus, or bilateral adnexa. The tumor was completely resected with a 2-cm margin of the greater omentum, which was divided at that point. The resection margins were confirmed to be microscopically negative (R0). Approximately 100 mL of blood-tinged ascitic fluid was present within the peritoneal cavity. The cytology of the peritoneal fluid showed no evidence of malignancy. The patient tolerated the surgical procedure well. His post-operative course was uneventful, with no immediate surgical complications. No adverse or unanticipated events related to the surgical intervention.

### 3.5 Pathological findings

Gross Examination: The specimen consisted of a well-encapsulated grayish-yellow to grayish-brown mass measuring 10.5×7.0×6.5cm. This discrepancy from the radiographic size and intraoperative sizes was attributed to deformation and hemorrhagic content. Serial sectioning revealed a heterogeneous appearance with hemorrhage and a predominantly solid, grayish-brown cut surface with a soft consistency. A focal area measuring 3.3 × 2.5 × 1.5 cm was noted, displaying a gray-white cut surface with a firm consistency.

**Figure 3:**
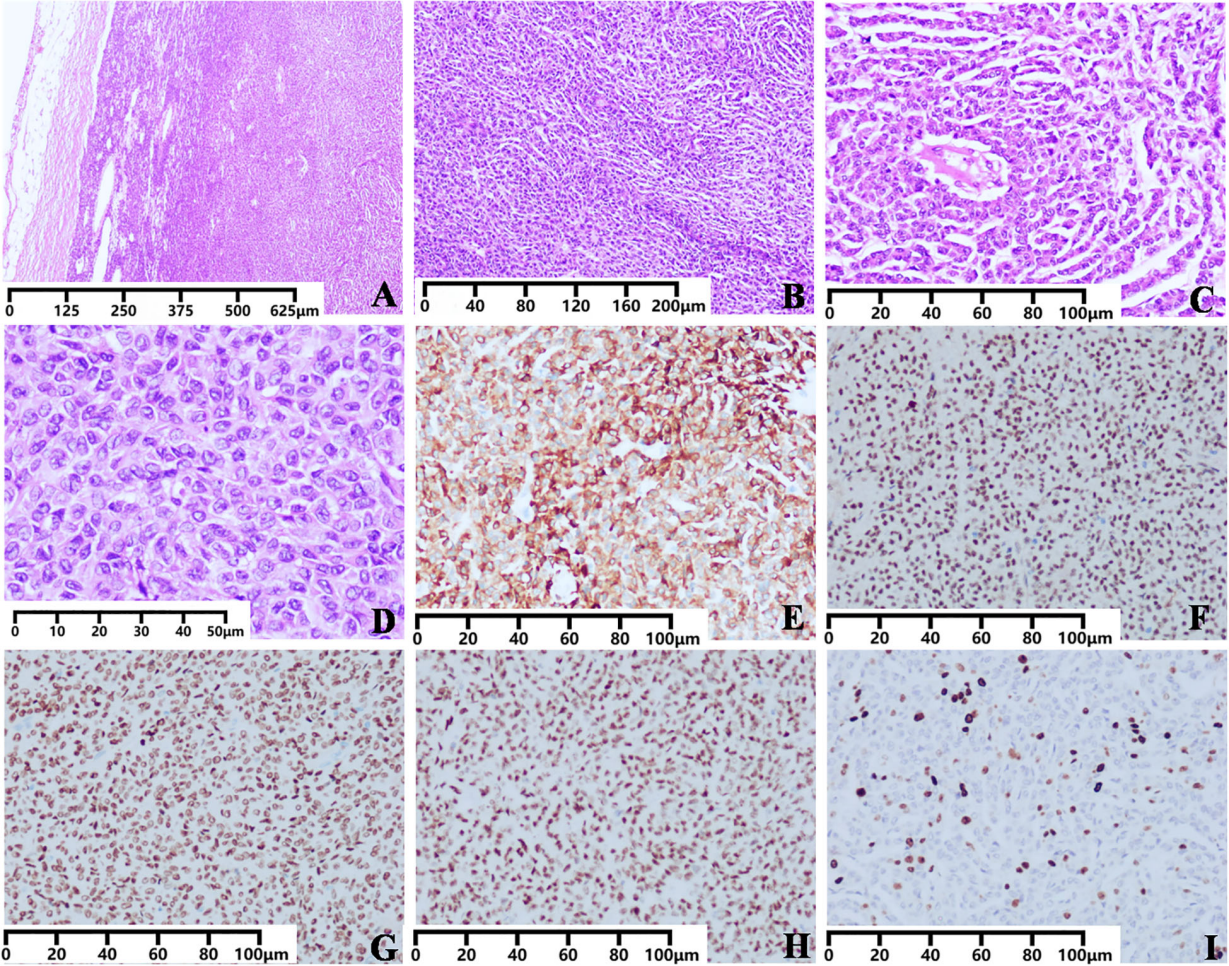
Pathological characteristics of the extraovarian adult granulosa cell tumor of the greater omentum. **(A–D)** Photomicrograph of the tumor (H&E stain). **(A)** The tumor is encapsulated (H&E stain, 40×magnification, Scale bar: 625 μm). **(B)** Neoplastic cells are arranged in cord-like and ribbon-like patterns (H&E stain, 100×magnification, Scale bar: 200 μm). **(C)** Call-Exner bodies are visible (H&E stain, 200×magnification, Scale bar: 100 μm). **(D)** The tumor is composed of round, oval, or polygonal cells with ill-defined cytoplasmic borders imparting a syncytial appearance. The scant cytoplasm ranges from pale to eosinophilic. Nuclei are round, oval, or angular with finely dispersed chromatin and inconspicuous nucleoli. Longitudinal nuclear grooves and coffee-bean shaped nuclei are present(H&E stain, 400×magnification, Scale bar: 50 μm). **(E–I)** Photomicrograph of immunohistochemical staining. Immunohistochemistry staining is positive for α-inhibin **(E)**, SF-1 **(F)**, FOXL2 **(G)**, WT-1 **(H)**. **(I)** Ki-67 Proliferation Index was 20% (IHC stain, 200× magnification, Scale bar: 100 μm).

Histopathological examination of the formalin-fixed, paraffin-embedded (FFPE) sections revealed the tumor was encapsulated by a fibrous capsule (**Figure 3A**). Neoplastic cells were arranged in cords and trabeculae exhibiting a streaming (“watered-silk”) pattern (**Figure 3B**). Call-Exner bodies were identified (**Figure 3C**). Extensive hemorrhage and focal necrosis were present. The tumor was composed of round, oval, or polygonal cells with indistinct (syncytial-like) borders and scant pale-to-eosinophilic cytoplasm. Nuclei were round, oval, or angular, with fine chromatin and inconspicuous nucleoli. Nuclear grooves and characteristic “coffee-bean” nuclei were observed (**Figure 3D**). Mitotic figures were counted in 50 consecutive high-power fields (HPFs, 40×objective, field area = 0.237 mm²). The count was 1 mitoses per 10 HPF. Satellite tumor nodules were identified within the adjacent adipose tissue. The margins were free of neoplasia, confirming an R0 resection.

Immunohistochemical findings. The tumor cells were diffusely positive for α-inhibin (**Figure 3E**), SF1 (**Figure 3F**), FOXL2 (**Figure 3G**), WT1 (**Figure 3H**), and Vimentin. Focal positivity was observed for CD10, D2-40, and SMA. Calretinin showed focal weak positivity. Tumor cells were negative for CD117, CD34, DOG1, Desmin, S100, HMB45, STAT6, Arginase1, keratin20, keratin5/6, keratin7, PAX8, Synaptophysin (Syn), Thyroglobulin (TG), TTF1, Glypican3, Villin, and SDHB. BAP1 was retained. The immunohistochemical profile was negative for CD117, CD34, and DOG-1, ruling out a gastrointestinal stromal tumor. Additionally, the tumor cells were negative for Desmin (arguing against a myogenic tumor), S100 (excluding malignant melanoma and neurogenic tumor), HMB45 (excluding melanoma), and STAT6 (arguing against a solitary fibrous tumor). Staining for Arginase-1 and Glypican-3 was negative, which helps exclude metastatic hepatocellular carcinoma. The diagnosis of mesothelioma was considered, but the tumor was lack of keratin5/6 and strong calretinin expression, retained BAP1 expression. The lack of synaptophysin expression effectively excludes a neuroendocrine carcinoma, along with the absence of PAX8, which renders primary carcinomas of the thyroid, kidney, or ovaries unlikely. Further, the tumor was negative for thyroglobulin and TTF-1 (excluding thyroid carcinoma), TTF-1 (arguing against a lung primary), and Villin (which helps exclude a carcinoma of digestive tract origin). The overall immunophenotype was not consistent with any of the above entities. The Ki67 proliferation index was 20% (**Figure 3I**). A summary of the immunohistochemical profile is provided in **Table 1**.

Integrated Diagnosis: The integration of clinical laboratory findings, imaging findings, histological morphology, and immunophenotype supports the diagnosis of an extraovarian adult granulosa cell tumor (primary in the greater omentum).

### 3.6 Patient follow-up

The patient recovered uneventfully and was placed on a structured follow-up regimen. This included clinical evaluation and contrast-enhanced CT scans at 3, and 6 months post-operatively. At the most recent follow-up, 6 months after surgery, the patient remained asymptomatic with no clinical or radiological evidence of recurrence. No adverse or unanticipated events related to the follow-up regimen were recorded during the entire 6-month period. The patient did not require readmission or additional unplanned procedures.

### 3.7 Clinicopathological characteristics of extraovarian adult granulosa cell tumors

#### 3.7.1 Epidemiological and clinical presentation

Clinicopathological Characteristics of Extraovarian Adult Granulosa Cell Tumors were summarized in **Table 2**. A total of 30 cases of extraovarian granulosa cell tumor (including our case) were analyzed. The age of the patients ranged from 3.5 to 86 years, with a median age of 55 years. The peak incidence occurred in the fifth to sixth decades, with 12 cases (40.0%) occurring in patients aged 50–59 years. Clinically, the most common presentations were abdominal distension and pain, while a minority of patients presented with irregular uterine bleeding or were asymptomatic.

#### 3.7.2 Tumor location and imaging features

Anatomically, the retroperitoneum was the most frequent site, involved in 17 cases (56.7%). Other sites included the adrenal gland (3 cases, 10.0%), mesentery (3 cases, 10.0%), broad ligament (2 cases, 6.7%), greater omentum (2 cases, 6.7%), and fallopian tube (2 cases, 6.7%). Radiologically, the tumors typically presented as complex cystic-solid masses, often with features of hemorrhage, necrosis, or calcification.

#### 3.7.3 Laboratory findings

Hormone levels were assessed in 7 of 30 cases (23.3%). Preoperative elevation of inhibin or estradiol was observed in 3 cases (10.0% of total series), with postoperative normalization documented in all 3 cases. Specifically, in Case 6 (7), the preoperative estradiol level was 1029 pg/ml, decreasing to <20 pg/ml postoperatively. In Case 26 (27), the preoperative inhibin level was >1300 pg/ml, declining to 388 pg/ml after surgery. Case 23 (24) showed normal postoperative inhibin B (16 pg/ml) and estrogen (18 pg/ml) levels. In the remaining 23 cases (76.7%), data on hormone levels were unavailable.

Tumor marker profiles were reported in 11 cases (36.7%). CA19-9, CA125, and CEA were negative in 10 of these 11 cases (90.9%). One case (10) showed an isolated elevation of CA125 to 83.08 U/ml, while all other markers (CA199, CEA) were negative. AFP and β-hCG were consistently negative in all 8 cases where they were tested.

#### 3.7.4 Pathological and immunohistochemical features

Tumor size was reported in 27 cases, ranging from 1.5 cm to 21.5 cm, with a median size of 9–10 cm. The majority of tumors (12 cases, 44.4%) measured between 5–10 cm, while 8 cases (29.6%) measured 10–15 cm, 3 cases (11.1%) measured 15–20 cm, 3 cases (11.1%) exceeded 20 cm, and 2 cases (7.4%) were smaller than 5 cm.

Histopathological evaluation confirmed adult-type granulosa cell tumor in 29 cases (96.7%), with one juvenile-type case (Case 23, 3.3%) included. Immunohistochemistry data were available for 22 cases(73.3%), demonstrating consistent positivity for sex cord-stromal markers: α-inhibin was positive in 21 of 22 tested cases (95.5%), calretinin in all 8 tested cases (100%), and WT-1 in all 4 tested cases (100%). Vimentin was positive in 4 of 4 tested cases (100%), SF-1 in 2 of 2 tested cases (100%), and FOXL2 in 1 of 1 tested case (100%). In contrast, epithelial markers showed consistent negativity: EMA was negative in 16 of 16 tested cases (100%), and CK was negative in 6 of 6 tested cases (100%). Neuroendocrine markers were uniformly negative: CgA in 5 of 5 tested cases (100%) and Syn in 6 of 6 tested cases (100%). Ki-67 proliferation index was generally low in adult-type tumors (ranging from <1% to 15%), but notably high (70%+) in the single juvenile case (Case 23).

Molecular analysis was performed in only 3 cases (10.0%), identified *FOXL2* mutations in 2 of these 3 tested cases (66.7%), both of which were adult-type tumors.

#### 3.7.5 Treatment and follow-up

All patients were treated with surgical resection. Only one patient received adjuvant chemotherapy (carboplatin + paclitaxel), and one received cytostatic therapy. During follow-up, only one case of recurrence was reported at 36 months postoperatively, while follow-up data of the majority of patients was unavailable.

## 4 Discussion

Our case provides a comprehensive clinicopathological characterization of a rare primary extraovarian granulosa cell tumors located in greater omentum, successfully managed through a combined diagnostic and surgical approach. The findings underscore the critical role of integrating imaging, histology, and immunohistochemistry (IHC) to achieve a definitive diagnosis and guide appropriate management.

### 4.1 Comparison with existing literature

#### 4.1.1 Diagnostic work-up

The diagnostic challenge posed by primary extraovarian granulosa cell tumors is well-documented. In our case, the CT findings of a well-defined, heterogeneous mass were non-specific, overlapping with more common entities like gastrointestinal stromal tumor, mesothelioma or metastatic carcinoma. The difficulty in preoperative diagnosis stemmed from the patient’s non-specific clinical symptoms. Additionally, due to the tumor’s location, imaging studies revealed intratumoral hemorrhage, which precluded a CT-guided biopsy. The definitive diagnosis was ultimately established by the distinct morphological features—specifically, round, oval, or polygonal cells and Call-Exner bodies—coupled with a definitive IHC profile (α-inhibin +, SF1 +, FOXL2+, WT1+). This aligns with literature emphasizing that a broad IHC panel is indispensable for differentiating among tumors of the Greater Omentum, as morphological overlap is frequent.

Anatomically, all primary sites were within the abdominal cavity. The retroperitoneum was the most frequent location (17 cases, 56.7%), followed by the adrenal gland, mesentery (3 cases each, 10.0%), broad ligament, greater omentum, and fallopian tube (2 cases each, 6.7%). Based on this anatomical distribution, we postulate that these tumors are more likely to originate from the coelomic epithelium or mesonephric (Wolffian) remnants.

#### 4.1.2 Treatment and outcome

The primary management for localized tumors of this type remains complete surgical resection, while chemotherapy is advised only in advanced stages (31). Our patient underwent an R0 resection via a laparoscopic approach, which was followed by a recurrence-free period of 6 months. This outcome is consistent with studies reporting favorable prognoses following complete excision. The low Ki-67 index (20%) and minimal mitotic activity (1/50 HPF) observed in our case are histopathological parameters associated with less aggressive clinical behavior.

### 4.2 Strengths and limitations

The principal strength of this report lies in the detailed pathological documentation and structured clinical follow-up, providing a valuable reference for this rare entity. However, several limitations must be acknowledged. This is a single-case report from a single institution, which inherently limits the generalizability of our findings. The follow-up period, while complete, remains relatively short for a tumor with a potential for late recurrence. Furthermore, this study has several inherent limitations, including its retrospective nature and the lack of advanced molecular profiling. More critically, the scarcity of follow-up data (available for only 3 of 30 cases) for extra-ovarian granulosa cell tumors in the published literature severely hampers a definitive analysis of their biological behavior. Future larger-scale, prospective studies are warranted to address these gaps.

### 4.3 Clinical take-home messages

#### 4.3.1 Diagnostic imperative

The Greater Omentum masses require a systematic diagnostic approach. Core biopsy or surgical resection for comprehensive histopathological and IHC analysis is essential for a definitive diagnosis.

#### 4.3.2 Key differential diagnoses

The main differential diagnoses include lymphoma, neuroendocrine tumors, epithelial malignancies, mesothelioma or sarcomas on routine hematoxylin-eosin (H&E) staining, their characteristic immunoprofile—coupled with the pathognomonic *FOXL2* c.402C>G mutation—readily facilitates distinction from these neoplasms. A targeted IHC panel is critical to distinguish between these possibilities.

#### 4.3.3 Recommended follow-up

A reasonable surveillance protocol should include clinical evaluation(e.g.,serum inhibin levels) and cross-sectional imaging (e.g., CT Abdomen) were advised every 3 months for the first postoperative year (31), every 6–12 months for the postoperative 2–3 years, then annually thereafter, considering the risk of late recurrence.

#### 4.3.4 Actionable triggers

The development of new symptoms, such as abdomen pain or any new concerning findings on surveillance imaging should prompt immediate further investigation and re-evaluation of the management plan.

## 5 Conclusion

In summary, we report a rare case of primary extra-ovarian adult granulosa cell tumor arising in the greater omentum, which represents only the second reported case at this anatomical site. Comprehensive imaging, laboratory tests, and intraoperative exploration thoroughly excluded an ovarian primary. The diagnosis was confirmed by its characteristic histomorphology and immunophenotype. Given the metastatic potential and risk of late recurrence associated with granulosa cell tumors, all patients with primary extra-ovarian lesions should undergo long-term surveillance, including regular imaging and hormonal marker monitoring. However, due to the extreme rarity of this tumor, accurate prognostic assessment will require further investigation through larger cohort studies.

## Data Availability

The original contributions presented in the study are included in the article/supplementary material. Further inquiries can be directed to the corresponding author.
